# Supplementary material for: The Role of Sirtuin 3 in Radiation-Induced Long-Term Persistent Liver Injury
Source: Antioxidants (Basel). 2020 May 11;9(5):409. doi: 10.3390/antiox9050409 (PMC7278565; doi:10.3390/antiox9050409)
Supplement: Supplementary file 1 [file antioxidants-09-00409-s001.pdf]

Supplemental Material

Table S1: Primers used in single gene QRT-PCR

PRIMER	SEQUENCE
Procoll 1 fwd	CAG GCA AAC CTG GTG AAC A
Procoll 1 rev	CTC GCC AGG GAA ACC TCT
α-SMA fwd	GAG GCA CCA CTG AAC CCT AA
α-SMA rev	CAT CTC CAG AGT CCA GCA CA
TGF-β fwd	CCT GTC CAA ACT AAG GC
TGF-β rev	GGT TTT CTC ATA GAT GGC G
IL-6 fwd	GAC AAC TTT GGC ATT GTG G
IL-6 rev	ATG CAG GGA TGA TGT TCT G
IL-1β fwd	TGG ACC TTC CAG GAT GAG GAC A
IL-1β rev	GTT CAT CTC GGA GCC TGT AGT G
GAPDH fwd	TGT TGC CAT CAA TGA CCC CTT
GAPDH rev	CTC CAC GAC GTA CTC AGC G

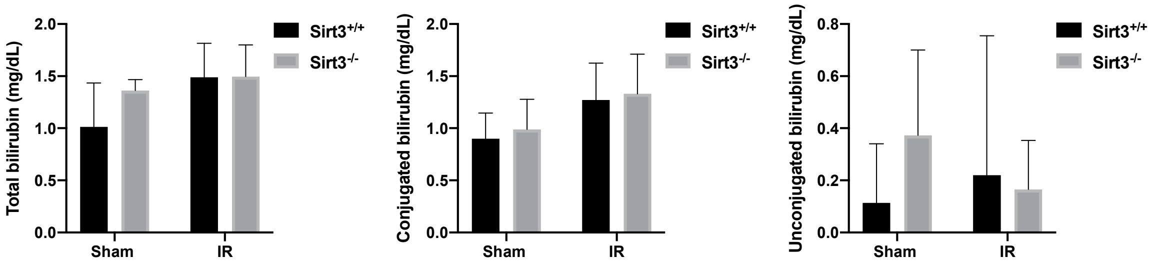

Figure S1. Exposure to 24 Gy liver only irradiation did not change the conjugated or unconjugated bilirubin levels in plasma obtained from Sirt3$^{+/+}$ or Sirt3$^{-/-}$ mice (n= 4-6).
